# Identification of the crosstalk among four types of adenosine‐related RNA modification in pan‐cancer

**DOI:** 10.1111/cas.15503

**Published:** 2022-08-14

**Authors:** Shichao Zhang, Yu Xiong, Weirong Wang, Erdong Zhang, Yan Gu, Yang Liu, Zhu Zeng, Fuzhou Tang, Yan Ouyang

**Affiliations:** ^1^ Key Laboratory of Infectious Immune and Antibody Engineering of Guizhou Province, Engineering Research Center of Cellular Immunotherapy of Guizhou Province Guizhou Medical University Guiyang China; ^2^ Immune Cells and Antibody Engineering Research Center of Guizhou Province, Key Laboratory of Biology and Medical Engineering Guizhou Medical University Guiyang China

## Abstract

Four types of A‐related RNA modification regulators interact with each other and even the crosstalk between the regulators could characterize the tumor immune microenvironment infiltration patterns, chemosensitivity, and cancer prognosis in patients with pan‐cancer.

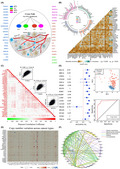

Dear Editor,

RNA modifications, mediated by “writers,” “readers,” and “erasers”, actively participate in tumorigenesis and cancer development, as well as immune dysregulation.^1^, ^2^, ^3^, ^4^ More than 170 types of RNA modifications have been identified, and several of these modifications could interact extensively.^5^ However, current research has mainly focused on single RNA modification types.^6^, ^7^, ^8^ Adenosine is the most prevalent modified RNA nucleotide, and its modifications include N1‐methyladenosine (m1A), N6‐methyladenosine (m6A), alternative polyadenylation (APA), and adenosine‐to‐inosine RNA editing (A‐I). Emerging evidence highlights competitively compensated interactions among these four types of modifications.^5^, ^9^ In particular, a previous study indicates that m6A methylase modulates A‐I.^10^ Nevertheless, the potential role of the crosstalk between the four A‐related RNA modification regulator types remains largely uncharacterized in human tumors.

To address this issue, we curated a catalog of 6, 20, 12, and 3 genes that function as m1A, m6A, APA, and A‐I regulators, respectively (Figure 1A). The transcriptome data among 11,080 pan‐cancer samples (33 cancer types: Table S1) were collected from The Cancer Genome Atlas for follow‐up analyses (Appendix S1: Materials and Methods). Almost all regulators had comparable expression levels in different cancer types (Figure S1). Next, we found that m6A regulators displayed relatively high mutation frequency, and genomic alterations were mutually exclusive across 41 regulators (Figure 1B). The copy number variations of these regulators were also prevalent in 33 tumor types (Figure 1E). Of note, the 41 regulators exhibited significant coexpression (Figure. 1C) and could even interact with each other (Figure 1F). We further observed that the expression of these regulators was associated with the activation or inhibition of multiple cancer‐related pathways (Figure S2), confirming that the four types of A‐related RNA modification regulators were functionally associated. In addition, based on their expression profiles, the healthy and tumor samples could be completely distinguished in 17 out of 33 tumors after excluding tumor types with the normal samples of <5 (Figure 1D and Figures S3–S4), implying that A‐related RNA modification patterns display potential for tumor diagnosis. Taken together, these results revealed that the crosstalk among the four types of A‐related RNA modification regulators plays an important role in cancer progression.

**FIGURE 1:**
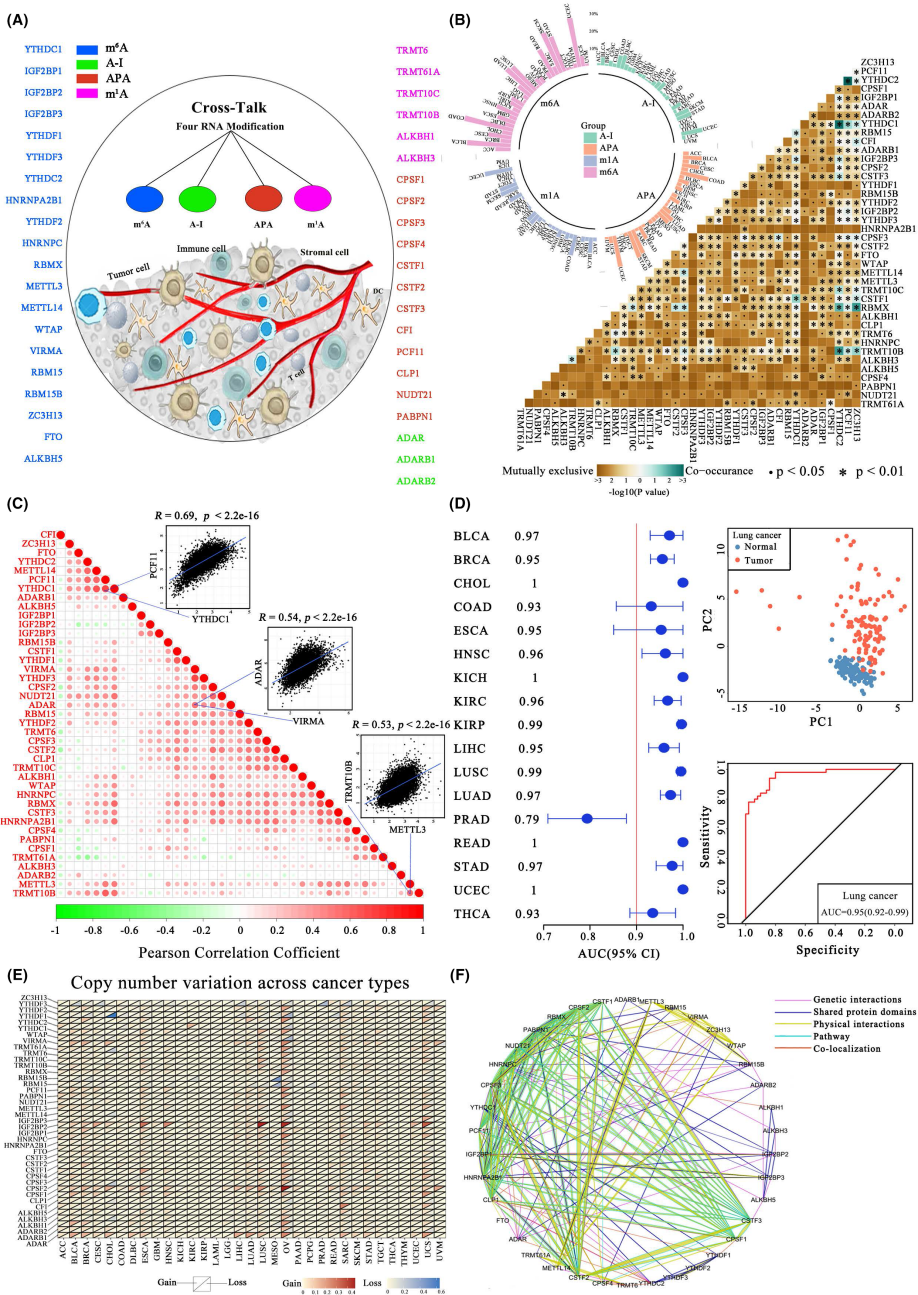
Crosstalk identified between the four types of A‐related RNA modification regulators. (A) N1‐methyladenosine (m1A), N6‐methyladenosine (m6A), alternative polyadenylation (APA), and adenosine‐to‐inosine RNA editing (A‐I) modification regulators and their potential crosstalk. (B) Mutation frequencies of 41 regulators and mutational correlation between different regulators across 33 cancer types. (C) Correlations among the expression of 41 regulators. (D) Principal component analysis based on the expression of 41 regulators to distinguish tumors from normal tissues in pan‐cancer (cancer types with more than five tumor–normal pairs included). For lung cancer, the area under the curve value for normal and cancer sample distinction was 0.95. (E) Copy number variation of 41 regulators across cancer types. (F) Protein–protein interactions among the 41 regulators

Based on weighted gene coexpression network analysis, we identified the hub genes of 33 cancer types from 41 A‐related RNA modification regulators (Figure 2A, and Table S2). Moreover, hub m6A regulators and other hub (m1A, APA, and A‐I) regulators were compared. The results showed that the number of the former was closely related to the total number of the latter in almost all tumor types (Figure 2B), which could be attributed to the crosstalk role of A‐related RNA modifications. Next, the integrated hub regulators of m6A, m1A, APA, and A‐I were defined as a module characteristic gene, which could characterize the RNA modification status. Based on the global expression of the module characteristic gene, a scoring model was constructed. Interestingly, tumors with high scores were remarkably negatively correlated with the abundance of almost all tumor‐infiltrating immune cell types across cancer types (Figure 2C). Subsequent immune response‐related signature analysis showed that all tumor types with increased scores were significantly enriched in stromal‐associated pathways, suggesting that the hub regulators might play important roles in the stroma activation (Figure 2D). Moreover, we evaluated the relationship of scores with the common chemotherapeutics efficacy among 33 cancer types, and the results indicate that the score model could potentially predict chemosensitivity (Figure S5). Thus, the crosstalk among the m6A, m1A, APA, and A‐I modification hub regulators could characterize the tumor immune microenvironment and chemosensitivity in pan‐cancer.

**FIGURE 2:**
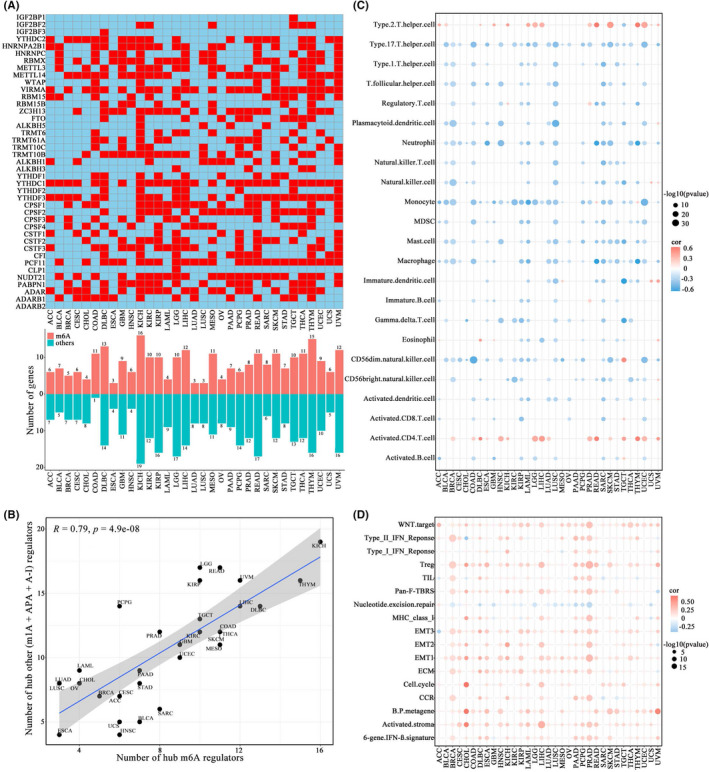
Identification and characterization of hub regulators in pan‐cancer. (A) Distribution map of the hub regulators of the four types of A‐related RNA modifications. The lower panel shows the number of hub N6‐methyladenosine (m6A) regulators and the total number of other hub (N1‐methyladenosine [m1A], alternative polyadenylation [APA], and adenosine‐to‐inosine RNA editing [A‐I]) regulators in each cancer type. (B) The association between the number of the hub m6A regulators and the total number of other hub (m1A, APA, and A‐I) regulators. (C) The correlation between the expression of the module characteristic gene and the ssGSEA scores of the tumor microenvironment cell infiltration across cancer types. (D) The correlation between the module characteristic gene expression and the score of stromal‐associated signatures across cancer types

To evaluate the prognostic value of the crosstalk among four types of A‐related RNA modification regulators in various cancers, a total of 18 hub regulators (all being the hub genes in at least 15 tumor types) were first excavated for constructing the A‐related RNA modification subtypes (Table S3). Based on the global expression profiles of these regulators, patients were then divided into two subtypes using an unsupervised clustering algorithm (Figures S6–S7). The obtained subtypes displayed a remarkable relationship with the overall survival in 13 out of 33 tumors (Figure S8 and S9A–B). However, no significant difference in survival was observed in almost all hematological tumors, possibly due to a high degree of heterogeneity between different tumor types. We further observed that the expression of most regulators significantly correlated with clinical parameters (Figure S9C–E), indicating that parameters containing regulators are involved in cancer progression. Taken together, the A‐related RNA modification pattern is of particular significance in guiding the prognostic tumor stratification and developing treatment strategies.

In summary, this study is the first to comprehensively analyze the role of the crosstalk among the four types of A‐related RNA modification regulators across 33 cancers. We demonstrated that these regulators could interact with each other and even the crosstalk between the regulators could characterize the tumor immune microenvironment infiltration patterns, chemosensitivity, and cancer prognosis in patients with pan‐cancer. This study provides new insights into epigenetic tumor regulation and effective therapeutic target development.

## CONFLICT OF INTEREST

The authors have no conflict of interest.

## FUNDING INFORMATION

The National Natural Science Foundation of China (31860244, 31760264, 32100442, and 31960139), the Science and Technology Foundation of Guizhou Province ([2021]172, [2020]1Z016, ZK[2021]025, [2020]1Y087, [2021]431, [2019]1275 and 19NSP002), and the Science and Technology foundation of Guizhou Health Committee (gzwjkj2019‐1‐037).

## Supporting information


Appendix S1
