# Supplementary material for: Identification of the crosstalk among four types of adenosine‐related RNA modification in pan‐cancer
Source: Cancer Sci. 2022 Aug 14;113(10):3633–6. doi: 10.1111/cas.15503 (PMC9530861; doi:10.1111/cas.15503)

**Identification of the crosstalk among four types of adenosine-related RNA modification in pan-cancer**

# Supplementary Information

[Materials and Methods 2](#_Toc66718366)

[Reference 4](#_Toc66718367)

[Table S1. Details of the 33 cancer types from the TCGA 5](#_Toc66718368)

[Table S2. Details of the hub genes of 33 cancer types from 41 A-related RNA modification regulators 6](#_Toc66718368)

[Table S3. Details of the tumor types corresponding to hub genes 8](#_Toc66718368)

[Figure S1. Expression profiles of the 41 A-related RNA modification regulators in 33 cancer types.. 9](#_Toc66718375)

[Figure S2. Relationship between the expression of A-related RNA modification regulators and cancer pathways. 1](#_Toc66718377)0

[Figure S3. Principal component analysis based on the expression of the 41 regulators to distinguish tumors (red dots) from normal tissues (blue dots) in 16 cancer types (cancer types with more than 5 tumor-normal pairs included). 1](#_Toc66718378)1

[Figure S4. The area under the curve (AUC) values to distinguish normal and cancer samples in 16 cancer types 1](#_Toc66718379)2

[Figure S5. Association between the global module characteristic gene expression and the common chemotherapeutics efficacy among 32 cancer types 1](#_Toc66718380)3

[Figure S6. Heat map exhibiting two subtypes identified by an unsupervised clustering algorithm based on the expression of 18 core regulators (each core gene was the hub gene of at least 15 tumor types) in 12 cancer types. 1](#_Toc66718381)4

[Figure S7. Proportion of the C1 and C2 subtypes across 13 cancer types 1](#_Toc66718382)5

[Figure S8. Survival analyses for patients grouped by an unsupervised clustering algorithm across 12 cancer types 1](#_Toc66718383)5

[Figure S9. Correlation between the four types of A-related RNA modification hub regulators and clinics in KIRC 1](#_Toc66718383)6

**Materials and Methods**

***Data acquisition***

We collected a total of six m1A, 20 m6A, 12 APA, and three A-I regulators^1-5^ and downloaded the entire transcriptome data among 11,080 pan-cancer samples (33 cancer types) from TCGA (https://tcga-data.nci.nih.gov/tcga/) database. Clinical information (including histologic grade, gender, age, and overall survival time), somatic mutation, and CNV data of the corresponding patients were retrieved from the University of California Santa Cruz genome browser (http://genome.ucsc.edu/). Patients with overall survival times of <90 days and without survival information were excluded. Table S1 shows the cancer types.

***Protein–protein interaction analysis across cancer types***

Using the GeneMANIA interaction database (<http://genemania.org>),^6^ we analyzed the protein–protein interactions among the six m1A, 20 m6A, 12 APA, and three A-I regulators and then used Cytoscape to visualize these interactions.^7^

***Gene Set Cancer Analysis***

We applied the available paired tumor–normal tissue expression data to evaluate the differential gene set expression profiles among 14 cancer types using the Gene Set Cancer Analysis (GSCA) web server.^8^ We also analyzed using the GSCA how the 41 A-related RNA modification regulators affect (activate or inhibit) cancer-related pathways. The GSCA includes 10 cancer-related pathways (apoptosis, cell cycle, DNA damage response, EMT, hormone AR, hormone ER, PI3K/AKT, RAS/MAPK, RTK, and TSC/mTOR) and regulators that activate or inhibit more than five cancer types.

***Identification of*** ***A-related RNA modification hub*** ***regulators based on coexpression network*** ***topology***

To identify hub m1A, m6A, APA, and A-I regulators for each cancer type, we first introduced the “module” concept from the WGCNA.^9-10^ The module expression levels were summarized as module eigengene using the moduleEigegenes function in the R package WGCNA. Then, we calculated the module membership that enabled the expression value relationship characterization of a given regulator and the module eigengene. Regulators with module memberships beyond 0.6 were defined as hub regulators. In each cancer type, we further calculated the overall hub regulators expression levels as an epigenetic module characteristic gene.

***Tumor microenvironment analysis across cancer types***

Single-sample gene set enrichment analysis (ssGSEA) was used to assess the level of each infiltrating immune cell type in the tumor microenvironment across cancer types,^11^ including activated B cells, CD4+ T cells, and CD8+ T cells, as well as gamma delta T cells, immature B cells, regulatory T cells, T follicular helper cells, type 1 T helper cells, type 17 T helper cells, type 2 T helper cells, activated DCs, CD56bright natural killer cells, CD56dim natural killer cells, eosinophils, immature DCs, macrophages, mast cells, MDSCs, monocytes, natural killer cells, natural killer T cells, neutrophils, and plasmacytoid DCs. Moreover, using GSVA,^12^ we analyzed the immune response-related signaling pathways and calculated the Pearson correlation coefficients between the hub regulator expressions and signaling pathways to determine regulators associated with pathway activation or inhibition.

***Unsupervised clustering and prognosis survival analysis***

On the basis of the hub regulator expression, we divided the tumor patients into two subtypes (C1 and C2) by the unsupervised clustering analysis across cancer types.^13^ For guaranteeing clustering stability, we performed 1000 iterations with Spearman distance and pltem = 0.8 using the PAM algorithm (“ConsensuClusterPlus” package). To further explore the C1 and C2 prognosis, we used the Kaplan–Meier method with a two-sided log-rank test. P-values of <0.05 were considered statistically significant.

***Drug sensitivity of 41 regulators across cancer types and clinical relevance of the hub regulators in KIRC***

We evaluated the relationship between the clinicopathological characteristics, such as the histologic grade, and diagnosis age, using the Wilcoxon signed-rank test. Moreover, we obtained the data on drug response from the Genomics of Drug Sensitivity in Cancer (<http://www.cancerrxgene.org/>downloads) and used Spearman’s correlation analysis to analyze the correlation between regulator expression and drug sensitivity. ^14^

**Reference**

1. Barbieri I, Kouzarides T. Role of RNA modifications in cancer. Nat Rev Cancer. 2020;20(6):303-322.
2. Li Y, Xiao J, Bai J, Tian Y, Qu Y, Chen X, et al. Molecular characterization and clinical relevance of m6A regulators across 33 cancer types. Mol Cancer. 2019 Sep 14;18(1):137.
3. Safra M, Sas-Chen A, Nir R, Winkler R, Nachshon A, Bar-Yaacov D, et al. The m1A landscape on cytosolic and mitochondrial mRNA at single-base resolution. Nature. 2017;551(7679):251-255.
4. Chen H, Yao J, Bao R, Dong Y, Zhang T, Du Y, et al. Cross-talk of four types of RNA modification writers defines tumor microenvironment and pharmacogenomic landscape in colorectal cancer. Mol Cancer. 2021;20(1):29.
5. Zhang B, Wu Q, Li B, Wang D, Wang L, Zhou YL. m6A regulator-mediated methylation modification patterns and tumor microenvironment infiltration characterization in gastric cancer. Mol Cancer. 2020;19(1):53.
6. Franz M, Rodriguez H, Lopes C, Zuberi K, Montojo J, Bader GD, et al. GeneMANIA update 2018. Nucleic Acids Res. 2018;46(W1):W60-W64.
7. Shannon P, Markiel A, Ozier O, Baliga NS, Wang JT, Ramage D, et al. Cytoscape: a software environment for integrated models of biomolecular interaction networks. Genome Res. 2003;13(11):2498-2504.
8. Liu CJ, Hu FF, Xia MX, Han L, Zhang Q, Guo AY. GSCALite: a web server for gene set cancer analysis. Bioinformatics. 2018;34(21):3771-3772.
9. Langfelder P, Horvath S. WGCNA: an R package for weighted correlation network analysis. BMC Bioinformatics. 2008;9:559.
10. Huang X, Zhang G, Tang T, Liang T. Identification of tumor antigens and immune subtypes of pancreatic adenocarcinoma for mRNA vaccine development. Mol Cancer. 2021;20(1):44.
11. Subramanian A, Tamayo P, Mootha VK, Mukherjee S, Ebert BL, Gillette MA, et al. Gene set enrichment analysis: a knowledge-based approach for interpreting genome-wide expression profiles. Proc Natl Acad Sci U S A. 2005;102(43):15545-15550.
12. Hänzelmann S, Castelo R, Guinney J. GSVA: gene set variation analysis for microarray and RNA-seq data. BMC Bioinformatics. 2013;14:7.
13. Diamond A, Schmuker M, Nowotny T. An unsupervised neuromorphic clustering algorithm. Biol Cybern. 2019;113(4):423-437.
14. Yang W, Soares J, Greninger P, Edelman EJ, Lightfoot H, Forbes S, et al. Genomics of Drug Sensitivity in Cancer (GDSC): a resource for therapeutic biomarker discovery in cancer cells. Nucleic Acids Res. 2013;41(Database issue):D955-D961.

**Table S1. Details of the 33 cancer types from the TCGA**

| **Cancer type** | **Abbreviation** | **Number of cancer samples** | **Number of normal samples** |
| --- | --- | --- | --- |
| Adrenocortical carcinoma | ACC | 79 | 0 |
| Bladder urothelial carcinoma | BLCA | 414 | 19 |
| Breast cancer | BRCA | 1,109 | 113 |
| Cervical squamous cell carcinoma and endocervical adenocarcinoma | CESC | 306 | 3 |
| Cholangiocarcinoma | CHOL | 36 | 9 |
| Colon adenocarcinoma | COAD | 480 | 41 |
| Lymphoid neoplasm diffuse large B-cell lymphoma | DLBC | 48 | 0 |
| Esophageal carcinoma | ESCA | 148 | 11 |
| Glioblastoma multiforme | GBM | 169 | 5 |
| Head and neck squamous carcinoma | HNSC | 502 | 44 |
| Kidney chromophobe | KICH | 62 | 24 |
| Kidney renal clear cell carcinoma | KIRC | 539 | 72 |
| Acute myeloid leukemia | LAML | 132 | 0 |
| Kidney renal papillary cell carcinoma | KIRP | 289 | 32 |
| Brain low-grade glioma | LGG | 529 | 0 |
| Liver hepatocellular carcinoma | LIHC | 332 | 50 |
| Lung adenocarcinoma | LUAD | 486 | 59 |
| Lung squamous cell carcinoma | LUSC | 440 | 49 |
| Mesothelioma | MESO | 77 | 0 |
| Ovarian serous cystadenocarcinoma | OV | 379 | 0 |
| Pancreatic adenocarcinoma | PAAD | 167 | 4 |
| Pheochromocytoma and paraganglioma | PCPG | 183 | 3 |
| Prostate adenocarcinoma | PRAD | 499 | 52 |
| Rectal adenocarcinoma | READ | 167 | 10 |
| Sarcoma | SARC | 251 | 2 |
| Skin cutaneous melanoma | SKCM | 471 | 1 |
| Stomach adenocarcinoma | STAD | 375 | 32 |
| Testicular germ cell tumor | TGCT | 156 | 0 |
| Thyroid carcinoma | THCA | 510 | 58 |
| Thymoma | THYM | 115 | 2 |
| Uterine corpus endometrial carcinoma | UCEC | 552 | 35 |
| Uterine carcinosarcoma | UCS | 56 | 0 |
| Uveal melanoma | UVM | 68 | 0 |

**Table S2. Details of the hub genes of 33 cancer types from 41 A-related RNA modification regulators**

| **Cancer types** | **m6A** | **m1A** | **A-I** | **APA** |
| --- | --- | --- | --- | --- |
| BRCA | METTL14, VIRMA, YTHDC1, YTHDC2, YTHDF3 | YTHDC1, YTHDF3 | ADAR | CPSF1, CPSF3, CPSF4, PABPN1 |
| COAD | HNRNPA2B1, HNRNPC, METTL14, RBM15, RBMX, VIRMA, WTAP, YTHDC1, YTHDC2, YTHDF1, YTHDF3 | TRMT10C, TRMT6, TRMT61A, YTHDC1, YTHDF1, YTHDF3 | ADARB1 | CPSF1, CPSF3, CSTF1, CSTF3, NUDT21 |
| ESCA | HNRNPC,RBMX,ZC3H13 | TRMT10B | ADARB1 | CSTF1, PCF11 |
| READ | HNRNPA2B1, HNRNPC, METTL14, RBMX, VIRMA, WTAP, YTHDC1, YTHDC2, YTHDF1, YTHDF3, ZC3H13 | ALKBH1, TRMT10C, TRMT6, TRMT61A, YTHDC1, YTHDF1, YTHDF3 | ADARB1 | CFI, CPSF1, CPSF2, CPSF3, CSTF1, CSTF2, CSTF3, NUDT21, PCF11 |
| STAD | METTL3, RBM15B, FTO, RBM15, VIRMA, YTHDF3, YTHDF1 | YTHDF1, TRMT61A, YTHDF3 | ADARB1 | CPSF2, CPSF4, NUDT21, PCF11 |
| HNSC | HNRNPA2B1, METTL14, RBM15, RBM15B, RBMX, YTHDC2 | TRMT10B |  | CPSF4, CSTF3, PABPN1 |
| PAAD | ALKBH5, METTL14, VIRMA, YTHDC1, YTHDC2, YTHDF3, ZC3H13 | ALKBH1, TRMT61A, YTHDC1, YTHDF3 | ADARB1 | CFI, CPSF2, NUDT21, PABPN1 |
| GBM | HNRNPA2B1, HNRNPC, METTL14, METTL3, RBM15B, RBMX, VIRMA, YTHDC1, YTHDC2, ZC3H13 | TRMT10B, TRMT10C, TRMT61A, YTHDC1 | ADAR | CPSF3, CSTF2, CSTF3, NUDT21, PABPN1, PCF11 |
| OV | FTO, HNRNPA2B1, METTL3, YTHDF1 | ALKBH1, TRMT10B, YTHDF1 | ADAR | CPSF1, CPSF2, CSTF1, NUDT21 |
| LUAD | VIRMA, YTHDC1, ZC3H13 | YTHDC1 | ADAR, ADARB1 | CPSF1, CPSF3, CPSF4, CSTF3, PCF11 |
| UCEC | ZC3H13, HNRNPC, METTL14, RBM15, RBMX, VIRMA, WTAP, YTHDC1, YTHDF3 | TRMT10C, TRMT6, YTHDC1, YTHDF3 | ADAR | CPSF2, CSTF2, CSTF3, NUDT21, PCF11 |
| KIRC | HNRNPA2B1, IGF2BP2, METTL14, METTL3, RBM15, VIRMA, YTHDC1, YTHDC2, YTHDF3, ZC3H13 | ALKBH1, TRMT10B, TRMT10C, YTHDC1, YTHDF3 | ADAR | CPSF1, CPSF2, CSTF2, NUDT21, PABPN1, PCF11 |
| LGG | HNRNPA2B1, METTL3, RBM15, RBMX, VIRMA, YTHDC1, YTHDC2, YTHDF2, YTHDF3, ZC3H13 | ALKBH3, TRMT10B, TRMT6, YTHDC1, YTHDF2, YTHDF3 | ADARB1 | CFI, CLP1, CPSF1, CPSF3, CSTF1, CSTF2, CSTF3, NUDT21, PABPN1, PCF11 |
| THCA | ALKBH5, FTO, HNRNPA2B1, IGF2BP2, METTL14, METTL3, RBMX, VIRMA, YTHDC1, YTHDC2, YTHDF3 | ALKBH3, TRMT10B, TRMT61A, YTHDC1, YTHDF3 | ADAR | CPSF2, CPSF4, CSTF1, NUDT21, PABPN1, PCF11 |
| LUSC | ALKBH5, HNRNPC, RBMX | ALKBH1, TRMT10B, TRMT10C |  | CPSF1, CPSF2, CPSF4, PABPN1, PCF11 |
| PRAD | FTO, METTL14, METTL3, VIRMA, YTHDC1, YTHDC2, YTHDF3, ZC3H13 | ALKBH3, TRMT10B, TRMT61A, YTHDC1, YTHDF3 | ADAR | CFI, CPSF1, CPSF2, NUDT21, PABPN1, PCF11 |
| SKCM | HNRNPA2B1, METTL14, METTL3, RBM15, RBMX, VIRMA, WTAP, YTHDC1, YTHDC2, YTHDF1, YTHDF3 | ALKBH1, TRMT10B, YTHDC1, YTHDF1, YTHDF3 | ADAR | CPSF1, CPSF2, CPSF3, CSTF2, NUDT21, PCF11 |
| BLCA | HNRNPA2B1, HNRNPC, METTL3, RBM15, RBMX, YTHDC1, YTHDF3 | YTHDC1, YTHDF3 | ADARB1 | NUDT21, PCF11 |
| LIHC | FTO, HNRNPA2B1, HNRNPC, METTL3, RBM15B, YTHDF2, RBMX, VIRMA, WTAP, YTHDC1, YTHDF1, YTHDF3 | TRMT10B, TRMT6, YTHDC1, YTHDF1, YTHDF2, YTHDF3 | ADAR | CPSF2, CPSF1, CPSF4, CSTF2, CSTF3, NUDT21, PCF11 |
| CESC | METTL14, RBMX, YTHDC1, YTHDC2, YTHDF3, ZC3H13 | ALKBH1, YTHDC1, YTHDF3 | ADAR, ADARB1 | CSTF1, NUDT21 |
| KIRP | HNRNPA2B1, METTL14, METTL3, RBM15B, RBMX, VIRMA, YTHDC1, YTHDF1, YTHDF3, ZC3H13 | TRMT10B, TRMT6, TRMT61A, YTHDC1, YTHDF1, YTHDF3 | ADAR | CPSF1, CPSF2, CPSF4, CSTF1, CSTF2, CSTF3, NUDT21, PABPN1, PCF11 |
| SARC | ALKBH5, METTL14, RBM15, RBMX, YTHDC1, YTHDC2, YTHDF3, ZC3H13 | YTHDC1, YTHDF3 |  | CSTF2, NUDT21, PABPN1, PCF11 |
| PCPG | FTO, METTL14, VIRMA, YTHDC1, YTHDF2, ZC3H13 | TRMT10B, TRMT10C, TRMT61A, YTHDC1, YTHDF2 | ADAR | CFI, CPSF2, CSTF1, CSTF2, CSTF3, NUDT21, PABPN1, PCF11 |
| TGCT | FTO, HNRNPC, IGF2BP1, IGF2BP2, RBM15, VIRMA, YTHDC1, YTHDF2, YTHDF3, ZC3H13 | ALKBH1, TRMT10C, TRMT6, YTHDC1, YTHDF2, YTHDF3 | ADAR, ADARB1 | CPSF2, CPSF4, CSTF2, PABPN1, PCF11 |
| THYM | FTO, HNRNPA2B1, HNRNPC, IGF2BP2, METTL14, METTL3, RBM15, RBM15B, RBMX, VIRMA, WTAP, YTHDC1, YTHDC2, YTHDF3, ZC3H13 | ALKBH3, TRMT10B, TRMT61A, YTHDC1, YTHDF3 | ADAR, ADARB1 | CFI, CPSF1, CPSF2, CPSF3, CPSF4, CSTF3, NUDT21, PABPN1, PCF11 |
| KICH | ALKBH5, FTO, HNRNPA2B1, HNRNPC, IGF2BP2, METTL14, METTL3, RBMX, VIRMA, WTAP, YTHDC1, YTHDC2, YTHDF1, YTHDF2, YTHDF3, ZC3H13 | TRMT10B, TRMT10C, TRMT6, TRMT61A, YTHDC1, YTHDF1, YTHDF2, YTHDF3 | ADAR, ADARB1 | CFI, CPSF1, CPSF2, CPSF3, CSTF2, CSTF3, NUDT21, PABPN1, PCF11 |
| ACC | HNRNPA2B1, RBM15, VIRMA, YTHDC1, YTHDC2, YTHDF3 | ALKBH1, YTHDC1, YTHDF3 | ADAR | CPSF3, CSTF1, CSTF3 |
| MESO | METTL3, METTL14, HNRNPA2B1, HNRNPC, IGF2BP2, RBM15B, RBMX, VIRMA, YTHDC1, YTHDF3, ZC3H13 | ALKBH1, TRMT6, YTHDC1, YTHDF3 | ADAR | CFI, CPSF2, CPSF4, CSTF2, NUDT21, PCF11 |
| UVM | HNRNPA2B1, HNRNPC, METTL14, METTL3, RBM15, RBMX, VIRMA, WTAP, YTHDC1, YTHDC2, YTHDF2, YTHDF3 | ALKBH1, TRMT10B, TRMT10C, YTHDC1, YTHDF2, YTHDF3 |  | CFI, CPSF1, CPSF2, CPSF3, CSTF1, CSTF2, CSTF3, NUDT21, PABPN1, PCF11 |
| DLBC | IGF2BP3, FTO, HNRNPA2B1, METTL14, METTL3, RBM15B, VIRMA, YTHDC1, YTHDC2, YTHDF1, YTHDF2, ZC3H13，YTHDF3 | TRMT10B, TRMT61A, YTHDC1, YTHDF1, YTHDF2, YTHDF3 | ADAR, ADARB1 | CFI, CPSF2, CPSF4, CSTF3, PABPN1, PCF11 |
| UCS | METTL14, VIRMA, YTHDC1, YTHDC2, YTHDF3, ZC3H13 | TRMT61A, YTHDC1, YTHDF3 |  | CFI, PCF11 |
| CHOL | METTL14, METTL3, VIRMA, YTHDC2 | TRMT10B | ADAR, ADARB1 | CPSF1, CPSF4, CSTF2, PABPN1, PCF11 |
| LAML | METTL14, RBMX, YTHDC1, ZC3H13 | TRMT10B, TRMT61A, YTHDC1 | ADAR | CPSF1, CPSF2, CPSF3, CSTF3, NUDT21 |

**Table S3. Details of the tumor types corresponding to hub genes**

| **Hub regulators** | **Tumor types** |
| --- | --- |
| ADAR | ACC, BRCA, CESC, CHOL, DLBC, DLBC, GBM, KICH, KIRC, KIRP, LAML, LIHC, LUAD, MESO, OV, PCPG, PRAD, SKCM, TGCT, THCA, THYM, UCEC |
| CPSF1 | BRCA, CHOL, COAD, KICH, KIRC, KIRP, LAML, LIHC, LUSC, OV, PRAD, READ, SKCM, THYM, UVM |
| CPSF2 | DLBC, KICH, KIRC, KIRP, LAML, LIHC, LUSC, MESO, OV, PAAD, PCPF, PRAD, READ, SKCM, STAD, TGCT, THCA, THYM, UCEC, UVM |
| CSTF2 | CHOL, GBM, KICH, KIRC, KIRP, LGG, LIHC, MESO, PCPG, READ, SARC, SKCM, TGCT, UCEC, UVM |
| CSTF3 | ACC, COAD, DLBC, GBM, HNSC, KICH, KIRP, LAML, LGG, LIHC, LUAD, PCPG, READ, THYM, UCEC, UVM |
| HNRNPA2B1 | ACC, BLCA, COAD, DLBC, GBM, HNSC, KICH, KIRC, KIRP, LGG, LIHC, MESO, OV, READ, SKCM, THCA, THYM, UVM |
| METTL14 | BRCA, CESC, CHOL, COAD, DLBC, GBM, HNSC, KICH, KIRC, KIRP, LAML, MESO, PAAD, PCPG, PRAD, READ, SARC, SKCM, THCA, THYM, UCEC, UCS, UVM |
| METTL3 | BLCA, CHOL, DLBC, GBM, KICH, KIRC, KIRP, LGG, LIHC, MESO, OV, PRAD, SKCM, TGCT, THCA, THYM, UVM |
| NUDT21 | BLCA, CESC, CAOD, GBM, KICH, KIRC, KIRP, LAML, LGG, LIHC, MESO, OV, PAAD, PCPG, PRAD, SARC, SKCM, STAD, THCA, THYM, UCEC, UVM |
| PABPN1 | BRAD, CHOL, DLBC, GBM, HNSC, KICH, KIRC, KIRP, LGG, LUSC, PAAD, PCPG, PRAD, SARC, TGCT, THCA, THYM, UVM |
| PCF11 | BLCA, CHOL, DLBC, ESCA, GBM, KICH, KIRC, KIRP, LGG, LIHC, LUAD, LUSC, MESO, PCPG, PRAD, READ, SARC, SKCM, STAD, TGCT, THCA, THYM, UCEC, UCS, UVM |
| RBMX | BLCA, CESC, COAD, ESCA, GBM, HNSC, KICH, KIRP, LAML, LGG, LIHC, LUSC, MESO, READ, SARC, SKCM, THCA, THYM, UCEC, UVM |
| TRMT10B | CHOL, DLBC, ESCA, GBM, HNSC, KICH, KIRC, KIRP, LAML, LGG, LIHC, LUSC, OV, PCPG, PRAD, SKCM, THCA, THYM, UVM |
| VIRMA | ACC, BRCA, CHOL, COAD, DLBC, GBM, KICH, KIRC, KIRP, LGG, LIHC, LUAD, MESO, PRAD, PCPG, PRAD, READ, SKCM, STAD, TGCT, THCA, THYM, UCEC, UCS, UVM |
| YTHDC1 | ACC, BLCA, BRCA, CESC, COAD, DLBC, GBM, KICH, KIRC, KIRP, LAML, LGG, LIHC, LUAD, MESO, PAAD, PCPG, PRAD, READ, SARC, SKCM, TGCT, THCA, THYM, UCEC, UCS, UVM |
| YTHDC2 | ACC, BRCA, CESC, CHOL, COAD, DLBC, GBM, HNSC, KICH, KIRC, LGG, PAAD, PRAD, READ, SARC, SKCM, THCA, THYM, UCS, UVM |
| YTHDF3 | ACC, BLCA, BRAD, CESC, COAD, DLBC, KICH, KIRC, KIRP, , LGG, LIHC, MESO, PAAD, PRAD, READ, SARC, SKCM, STAD, TGCT, THCA, THYM, UCEC, UCS, UVM |
| ZC3H13 | CESC, DLBC, ESCA, GBM, KICH, KIRC, KIRP, LAML, LGG, LUAD, MESO, PAAD, PCPG, PRAD, READ, SARC, TGCT, THYM, UCEC, UCS |

**Figure S1. Expression profiles of 41 A-related RNA modification regulators in 33 cancer types.**


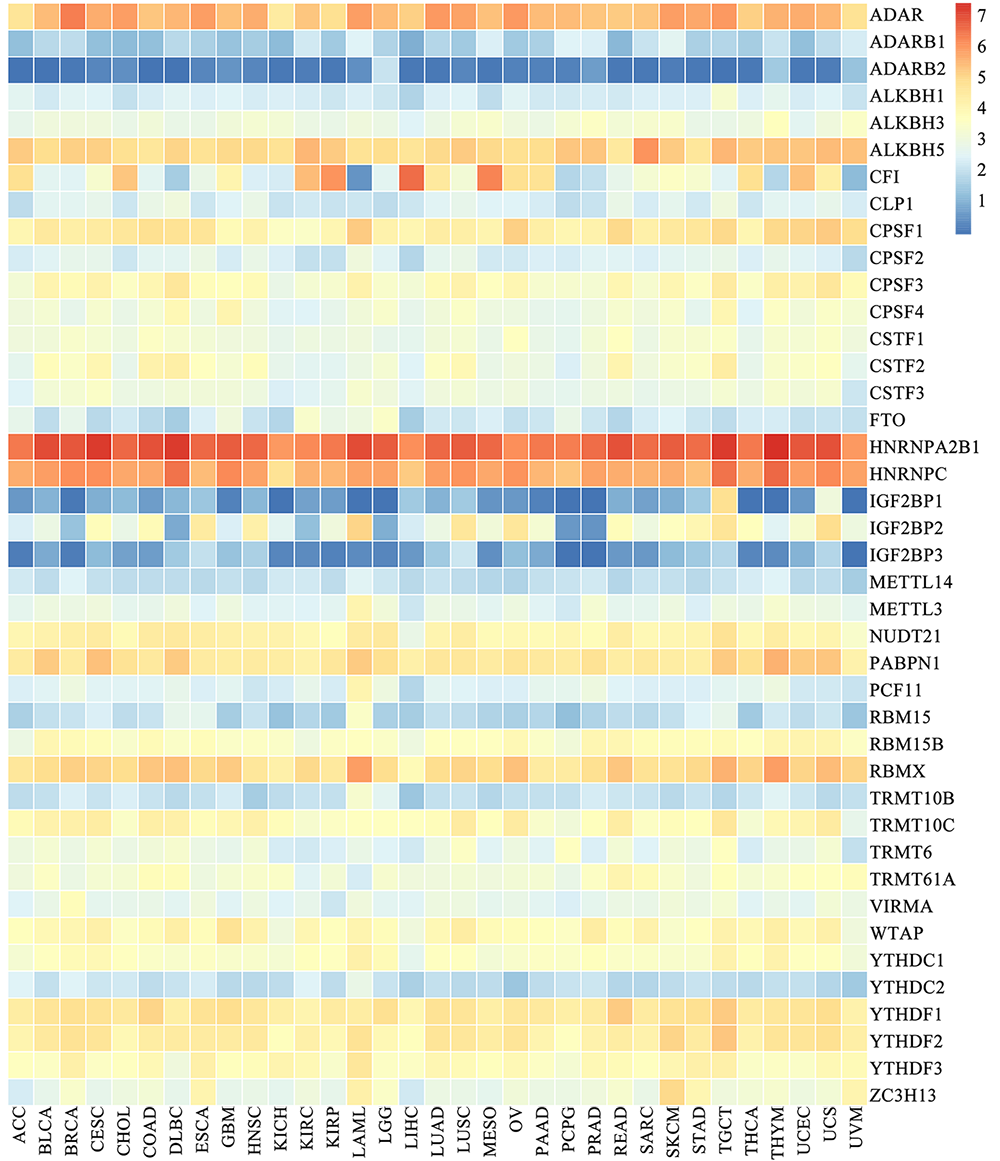


**Figure S2. Relationship between the expression of A-related RNA modification regulators and cancer pathways.**


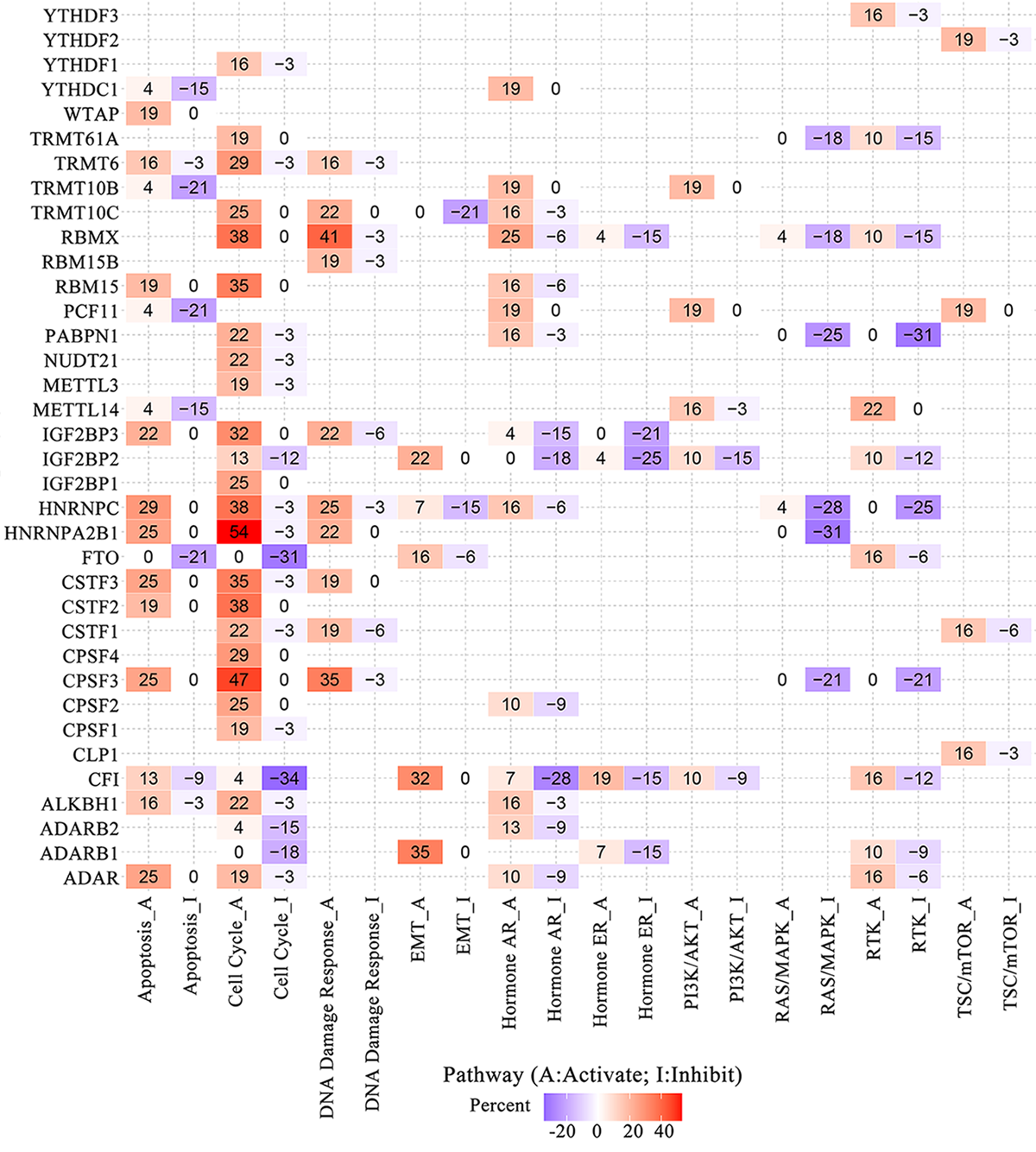


**Figure S3. Principal component analysis based on the expression of the 41 regulators to distinguish tumors (red dots) from normal tissues (blue dots) in 16 cancer types (cancer types with more than 5 tumor-normal pairs included).**


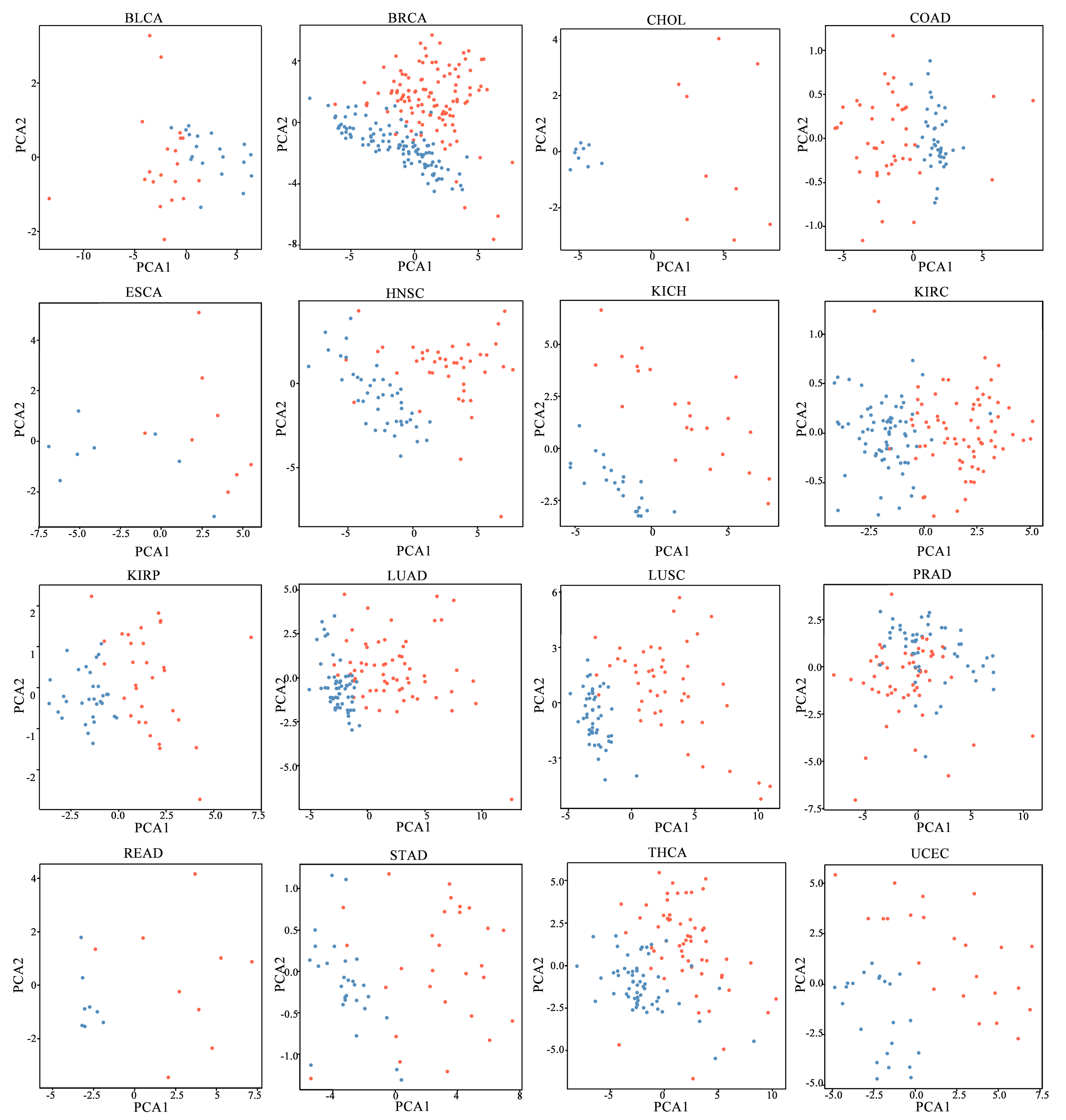


**Figure S4. The area under the curve (AUC) values to distinguish normal and cancer samples in 16 cancer types.**


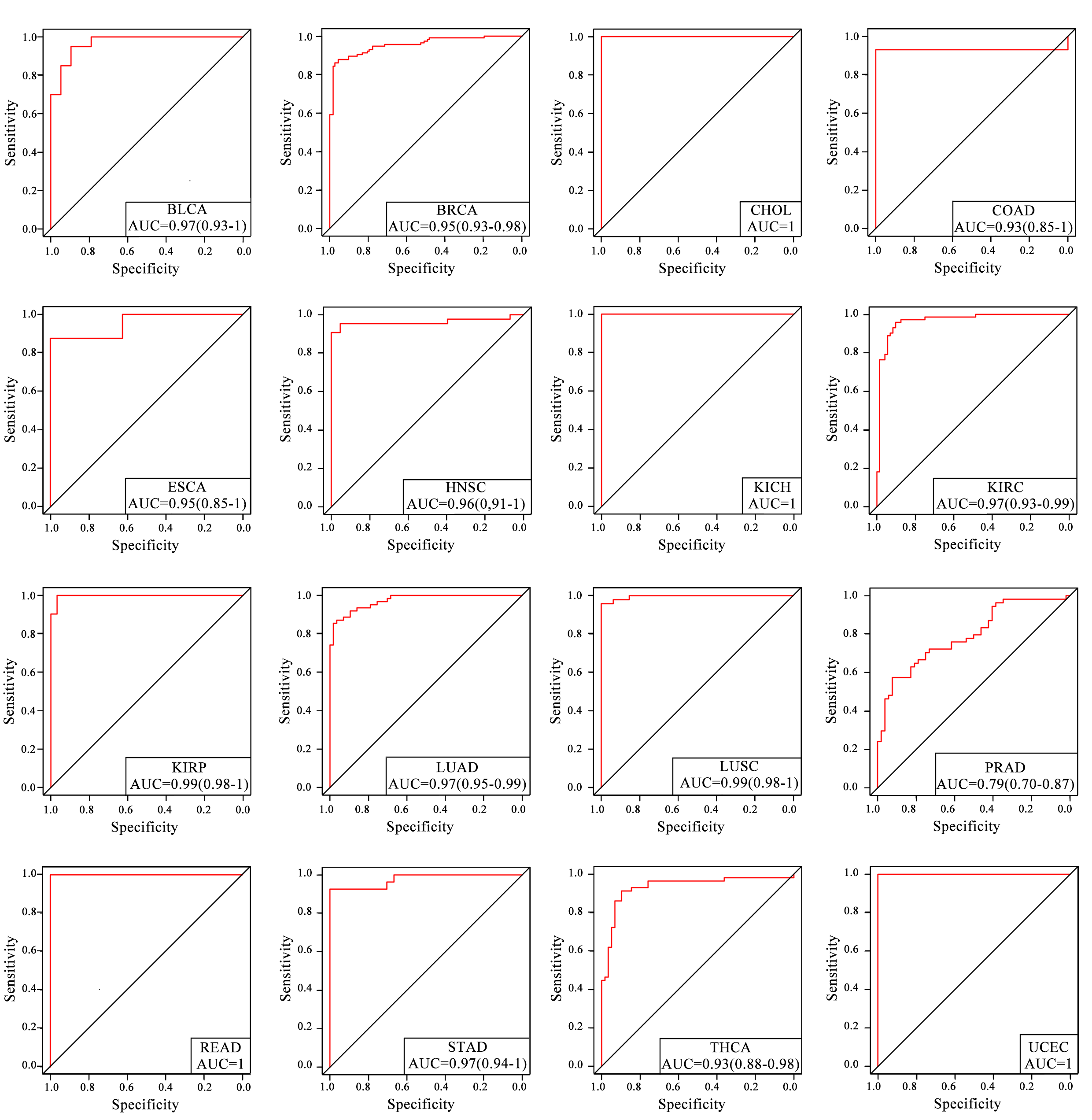


**Figure S5. Association between the global module characteristic gene expression and the common chemotherapeutics efficacy among 32 cancer types.**


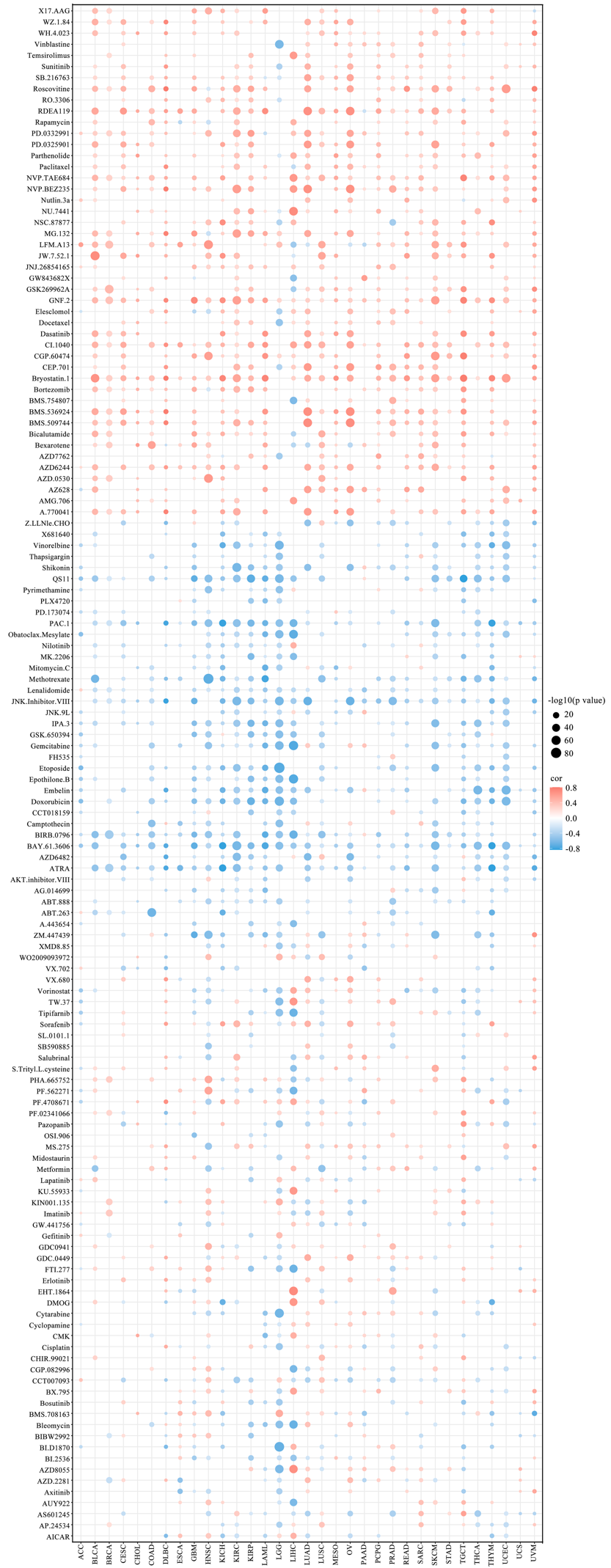


**Figure S6. Heat map exhibiting two subtypes identified by an unsupervised clustering algorithm based on the expression of 18 core regulators (each core gene was the hub gene of at least 15 tumor types) in 12 cancer types.**


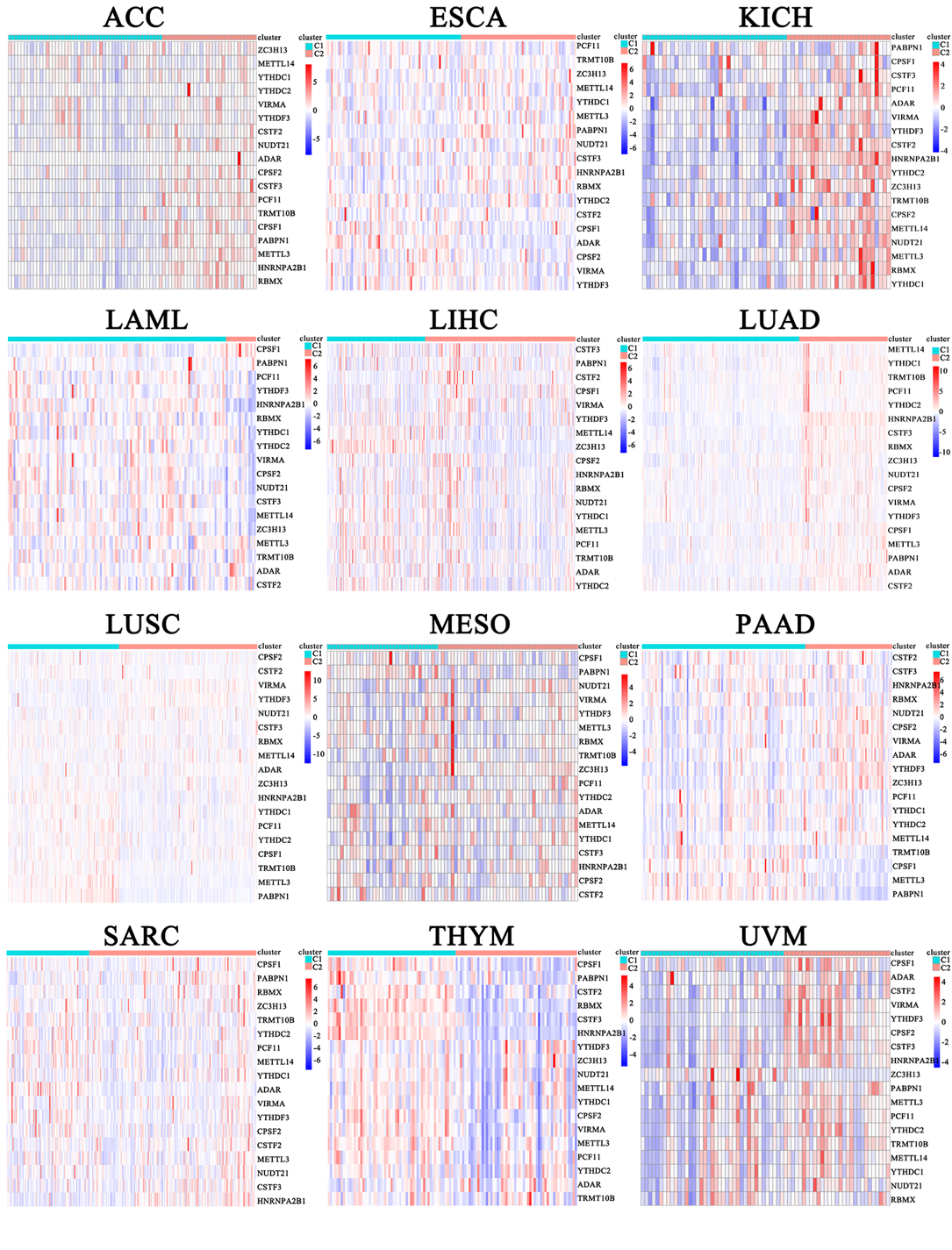


**Figure S7. Proportion of the C1 and C2 subtypes across 13 cancer types.**


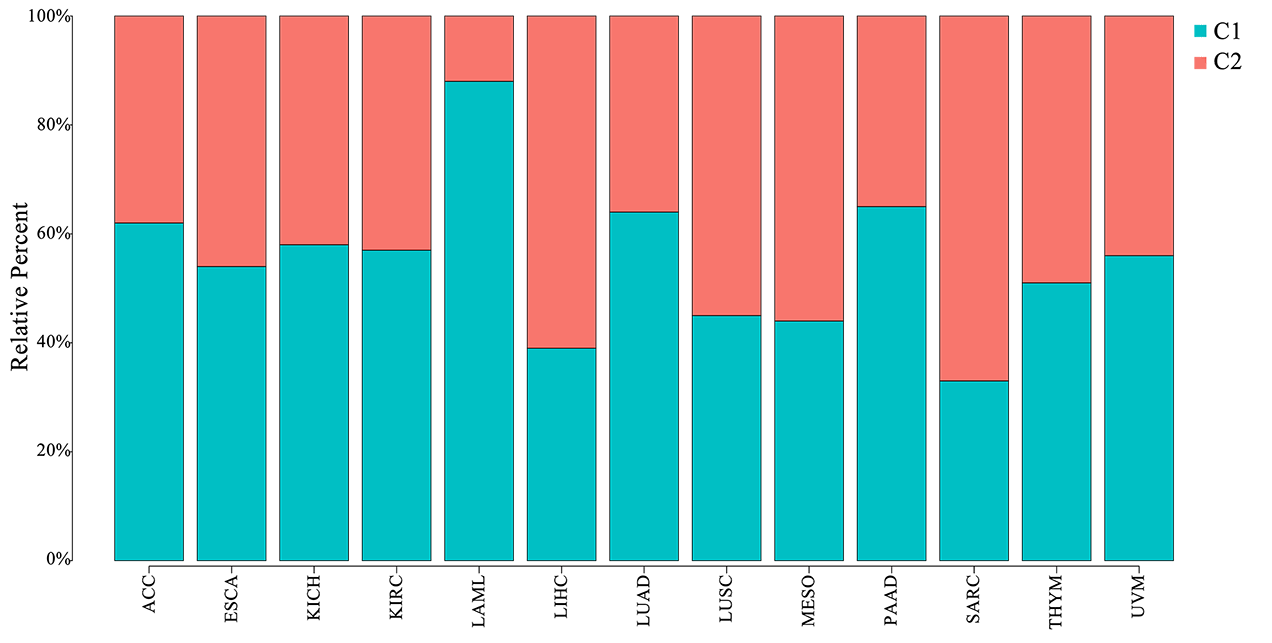


**Figure S8. Survival analyses for patients grouped by an unsupervised clustering algorithm across 12 cancer types.**


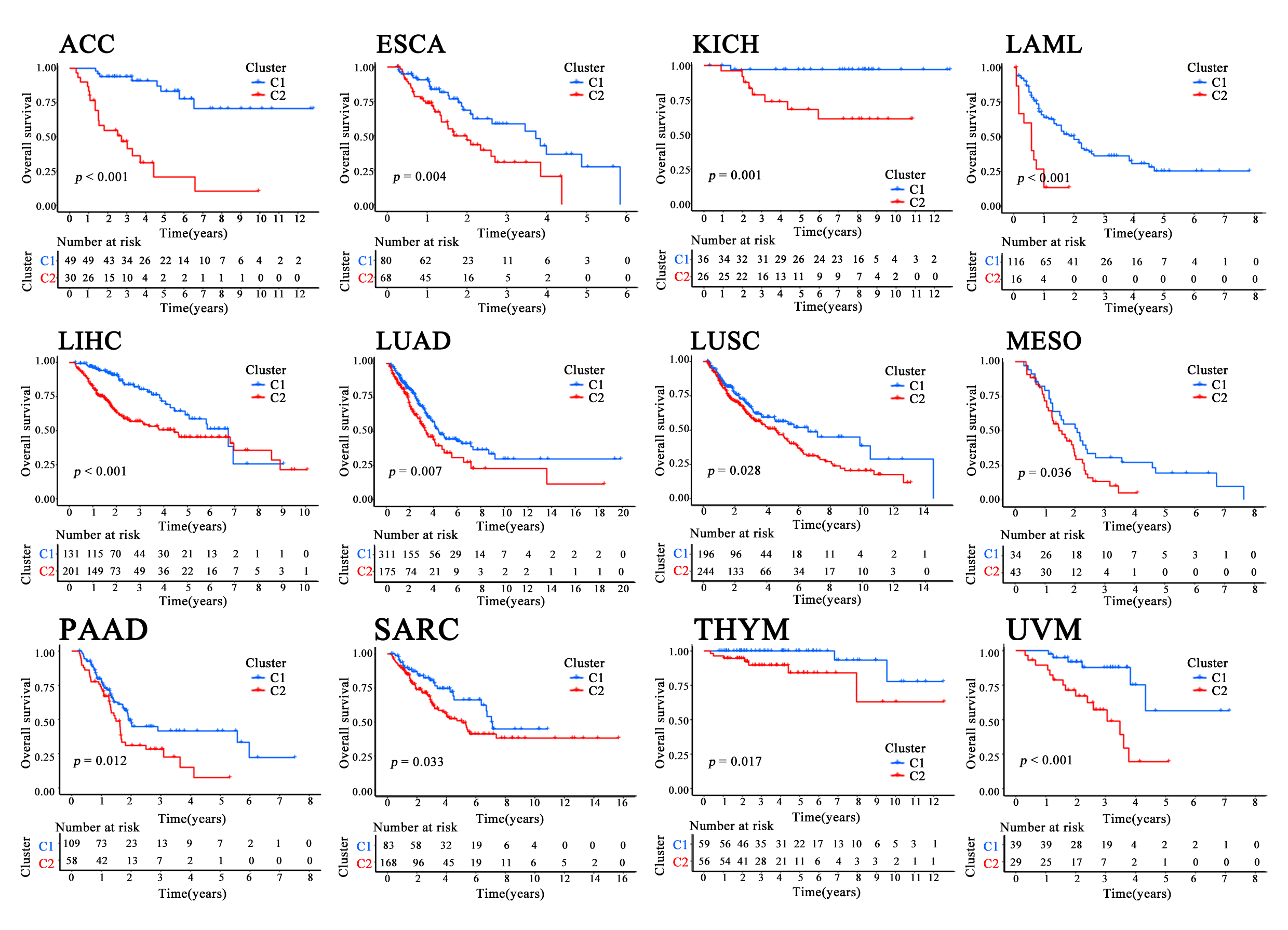


**Figure S9.** Correlation between the four types of A-related RNA modification hub regulators and clinics in KIRC. **A** Heat map exhibiting two subtypes identified using an unsupervised clustering algorithm based on the expression of 18 core regulators (Each core gene was the hub gene of at least 15 tumor types). **B** Survival analyses for the C1 and C2 subtypes based on 502 patients (log-rank test). **C** Association between certain hub regulators and histologic grade, and patient age (ANOVA). **D-E** Kyoto Encyclopedia of Genes Genomes pathway analysis for the C1 (**D**) and C2 (**E**) subtypes.


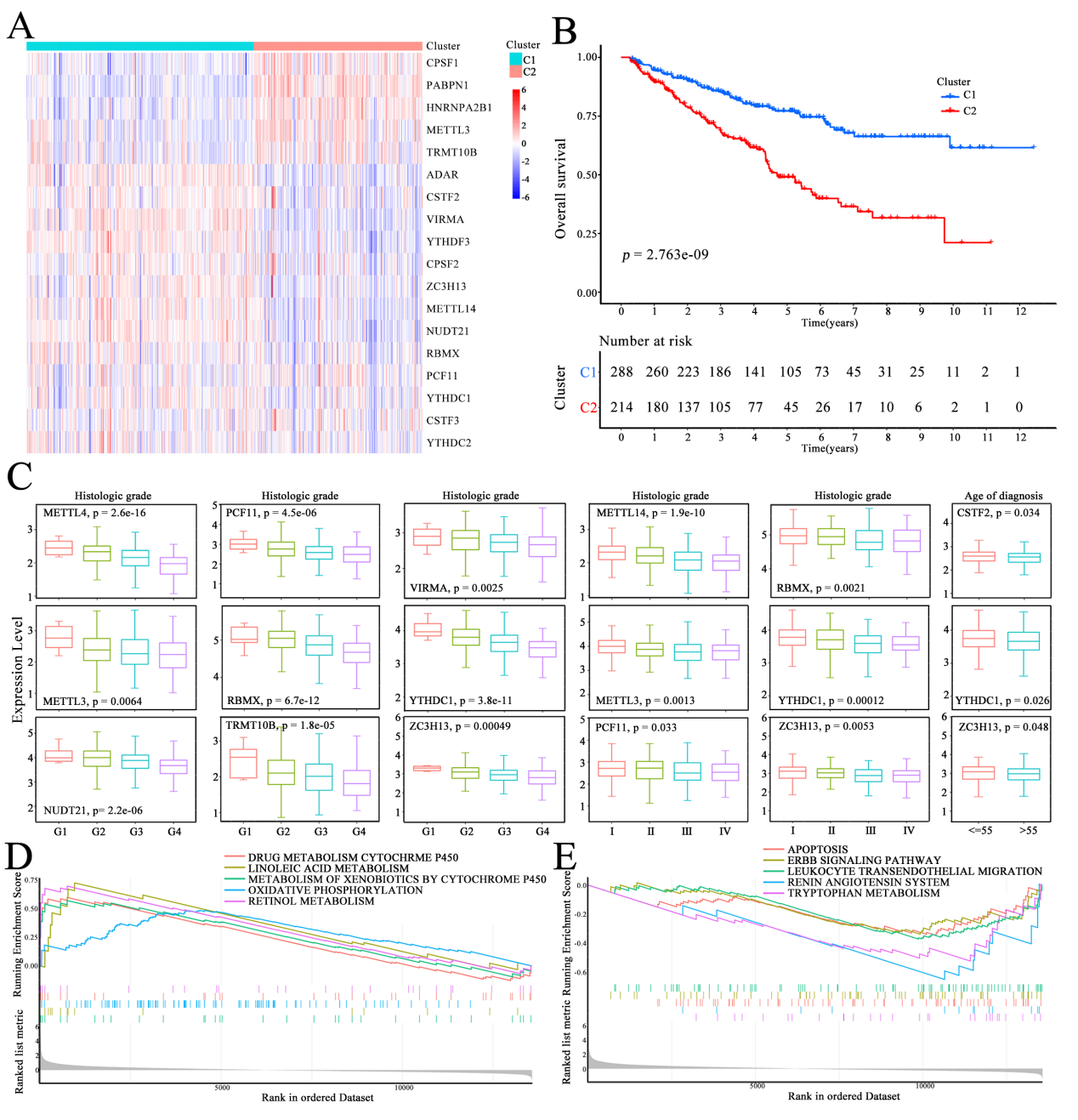

Supplement: Supplementary file 1 — Appendix S1 [file CAS-113-3633-s001.docx]
